# Coherence and pulse duration characterization of the PAL-XFEL in the hard X-ray regime

**DOI:** 10.1038/s41598-019-39765-3

**Published:** 2019-03-01

**Authors:** Kyuseok Yun, Sungwon Kim, Dongjin Kim, Myungwoo Chung, Wonhyuk Jo, Hyerim Hwang, Daewoong Nam, Sangsoo Kim, Jangwoo Kim, Sang-Youn Park, Kyung Sook Kim, Changyong Song, Sooheyong Lee, Hyunjung Kim

**Affiliations:** 1 Department of Physics, Sogang University, Seoul, 04107 Korea; 2 Korea Research Institute of Standards and Science, Daejeon, 34113 Korea; 3 Pohang Accelerator Laboratory, Pohang, 37673 Korea; 4 Department of Physics, Pohang University of Science and Technology, Pohang, 37673 Korea

## Abstract

We characterize the spatial and temporal coherence properties of hard X-ray pulses from the Pohang Accelerator Laboratory X-ray Free Electron Laser (PAL-XFEL, Pohang, Korea). The measurement of the single-shot speckle contrast, together with the introduction of corrections considering experimental conditions, allows obtaining an intrinsic degree of transverse coherence of 0.85 ± 0.06. In the Self-Amplified Spontaneous Emission regime, the analysis of the intensity distribution of X-ray pulses also provides an estimate for the number of longitudinal modes. For monochromatic and pink (i.e. natural bandwidth provided by the first harmonic of the undulator) beams, we observe that the number of temporal modes is 6.0 ± 0.4 and 90.0 ± 7.2, respectively. Assuming a coherence time of 2.06 fs and 0.14 fs for the monochromatic and pink beam respectively, we estimate an average X-ray pulse duration of 12.6 ± 1.0 fs.

## Introduction

X-ray Free Electron Lasers (XFELs) deliver orders of magnitude more brilliant, nearly fully transversely coherent and ultrashort X-ray pulses than previously available at synchrotron storage-ring based sources^1,2^. These unique properties enable probing complex structural dynamics down to femtosecond time-scales by means of optical-pump X-ray-probe scattering^3,4^, X-ray photon correlation spectroscopy^5–7^, and single-pulse coherent diffraction imaging^8–10^. In this regard, many XFEL experiments, therefore, rely on the coherence properties of the radiation. The coherence property of X-ray beams can be characterized by the speckle contrast or the visibility of coherent diffraction patterns. Their value range between zero (no coherence) up to unity (full coherence).

For instance, by utilizing the short pulse duration and nearly full transverse coherence of the beam, high-contrast speckles have been measured from atomic-scale ordering^11^. Such properties allow, for example, capturing instantaneous snap-shots of ferromagnetic, nanoscale spin order via single-pulse resonant X-ray holography techniques^12^. In combination with the recent developments of generating two XFEL pulses separated in time (i.e. either generated by the FEL itself or with hard X-ray split-delays optics^13–15^) these sources offer the promise for probing dynamics in amorphous and disordered systems on length- and time-scale of interest by means of X-ray Speckle Visibility Spectroscopy^13,16^.

The coherence properties of XFEL radiation differ from that of optical lasers because of the initial electron beam shot-noise that gets amplified during the Self-Amplified Spontaneous Emission (SASE) process^17^. While the beam is expected to be nearly fully transversely coherent, in which the output radiation is dominated by a single spatial mode near SASE saturation, each XFEL pulse carries multiple temporal modes. The energy distribution among these modes varies randomly for subsequent X-ray pulses resulting in intensity fluctuations. Such behavior is strongly coupled to the details of the operational parameters of the accelerator. Therefore, characterizing the single-shot and average coherence properties of XFEL pulses is essential for these new light sources to realize their full potential.

There have been several attempts using other methods to characterize the coherence of SASE XFEL sources. In the soft X-ray regime, Young’s double-slit and pinhole experiments^18–20^ were used to evaluate the transverse coherence. The visibility from two time-delayed partial beams obtained by wavefront beam splitting in an autocorrelator was employed to quantify its longitudinal coherence^21^. However, extending to the hard X-ray regime such measurements with double slits and pinholes presents some difficulties. The degree of coherence has however been characterized using the speckle contrast or visibility from coherent diffraction patterns and intensity fluctuations of successive X-ray pulses. The scattering in the small- and wide- angle scattering regimes are thus analyzed to obtain the transverse and longitudinal coherence properties of the beam, respectively^22–25^.

The speckle contrast *β* can be characterized by fitting the intensity probability distribution with a negative binomial function below^26^:

1 $$p(I)=\frac{{\rm{\Gamma }}(I+M)}{{\rm{\Gamma }}(M){\rm{\Gamma }}(I+1)}{(1+\frac{M}{\langle I\rangle })}^{-I}{(1+\frac{\langle I\rangle }{M})}^{-M},$$

where $I$ is the single shot intensity of scattered X-rays, $\langle I\rangle$ the mean intensity, ${\rm{\Gamma }}(x)$ the Gamma function, and $M$ the number of modes. The contrast *β* can be interpreted as a measurement of the ratio between the scattering and the coherence volumes and is related to the number of modes *M* by $\beta =1/\sqrt{M}$. Such technique was successfully used to evaluate the coherence properties of other hard X-ray FELs such as LCLS^22,23^ and SACLA^24^ for which the degree of the transverse coherence was measured to be 0.94 ± 0.03 at 8.96 keV and 0.79 ± 0.09 at 8.0 keV, respectively. However, a recent SACLA study^27^ which accounts for the exact sampling of coherent diffraction patterns in combination with recovering missing parts of the diffraction data by multiplying with a Gaussian mask, obtained a degree of coherence of 0.997 ± 0.001 at 5.5 keV. Similar corrections involving the speckle size have also been done in laser speckle contrast analysis^28^.

In this work, the coherence of the PAL-XFEL^29^ X-ray beam is determined at 9.73 keV by analyzing the speckle contrast from coherent diffraction pattern produced by illuminating silica gels and gold nanoparticles with X-ray pulses focused at the sample position using Kirkpatrick-Baez (KB) mirrors^30^. Here, we further address the effects of spatial averaging which reduce the measurable speckle contrast and obtain an estimate for the intrinsic degree of spatial coherence based on such corrections. We also provide an estimate for the longitudinal coherence and the pulse duration of the PAL-XFEL beam by analyzing beam intensity fluctuations both with (i.e. monochromatic) and without (i.e. pink) a monochromator. The pulse duration obtained from the analysis of the intensity distributions of the beam is 12.6 ± 1.0 fs.

## Results and Discussion

**Figure 1:**
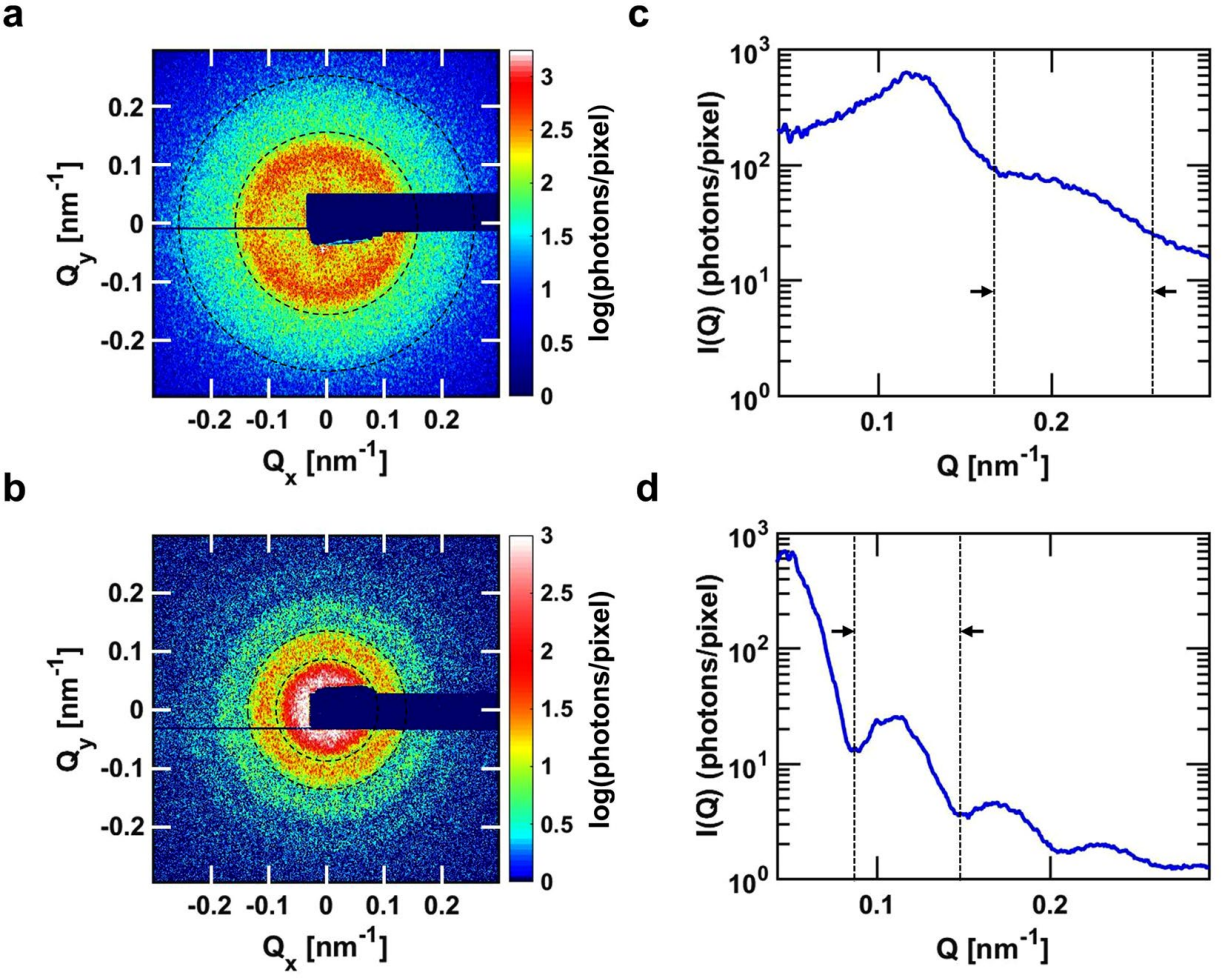
Single-shot speckle patterns in SAXS geometry. Single-shot Small Angle coherent X-ray Scattering patterns of (**a**) the silica gel and (**b**) gold nanoparticles and their azimuthally averaged single-shot intensities I(Q) displayed in (**c**,**d**), respectively. The contrast analysis was performed for pixels located in ROIs highlighted in between the dashed lines in (**a**–**d**); which translates into Q = 0.211 ± 0.071 nm^−1^ in (**a**,**c**) and Q = 0.112 ± 0.027 nm^−1^ in (**b**,**d**).

Single shot speckle patterns measured from a colloidal silica gel and gold nanoparticles are displayed in Fig. 1(a,b) respectively. The experimental details are described in Methods. The mean diameters of silica and gold nanoparticles are 50.4 ± 10.6 nm and 101 ± 11.8 nm, respectively (Supplementary Fig. S1). The mean scattering intensity per shot I(Q) is obtained by azimuthally averaging the speckle patterns and are displayed in Fig. 1(c,d). The concentric rings observed in the scattering patterns in Fig. 1(a,b) correspond to the oscillations observed in I(Q) and are typical from a spherical form factor. These rings are further decorated by grainy features also known as speckles and are typical of a coherent scattering pattern. The speckle contrast is evaluated in concentric regions of interests (ROIs) by fitting the intensity distribution from the pixels contained in these areas with the intensity distribution described in Eq. (1). We used ROIs consisting of pixel elements that form an annulus of radius Q = 0.211 ± 0.071 nm^−1^ for the silica gel and Q = 0.112 ± 0.027 nm^−1^ for the gold nanoparticles as indicated by the areas defined by the dashed lines in Fig. 1.

**Figure 2:**
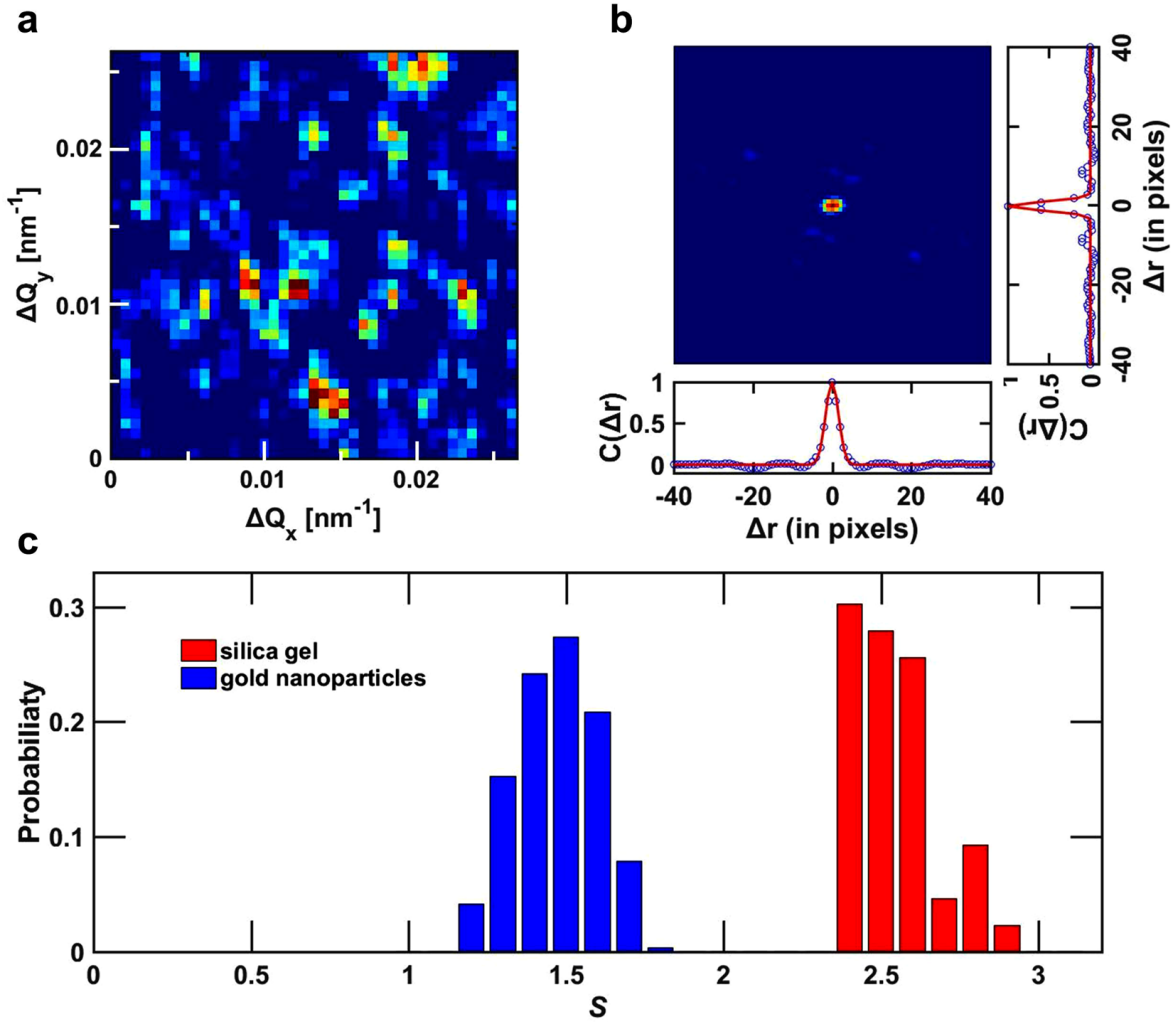
Spatial autocorrelation of a speckle pattern and deduced speckle size/area. (**a**) Zoomed-in view of the single shot speckle pattern displayed in Fig. 1(**a**). (**b**) Two-dimensional spatial auto-correlation of the speckle pattern in (**a**) Line-cuts along the vertical and horizontal directions (symbol) through the center with fits with a Gaussian profile (solid line). The detector pixel size is 50 × 50 μm^2^. (**c**) Histogram of the distribution of speckle size *S* in units of the pixel size as defined by Eq. (2) for both the silica gel (red) and the gold nanoparticles (blue).

To reflect the influence of the speckle size to the contrast in the analysis, the speckle size is determined for each shot. Figure 2(a) displays a zoomed view from the single shot speckle pattern in Fig. 1(a), from which discernable speckles are observed. The individual speckle size is obtained by calculating the spatial autocorrelation function displayed in two dimensions in Fig. 2(b). Line-cuts in the horizontal and vertical directions are then fitted by a Gaussian function as displayed in Fig. 2(b) which yield a Full Width at Half Maximum (FWHM) speckle size of 192 μm × 123 μm (H × V) for the silica gel sample. To first order, the spatial size of individual speckles *D* is approximated with $D=\lambda L/d$, where *L* is the distance between the sample and the detector, λ the wavelength, and *d* the beam size at the sample location. The beam size obtained for the data in Fig. 2(b) gives an FWHM of 2.5 μm × 3.9 μm (H × V) consistent with the result obtained from an independent knife-edge scan measurement of 2.5 μm × 3.5 μm (H × V) (data not shown). However, we observe that *d* fluctuates from shot to shot. This can be attributed to changes in the optical path (beam positions, pointing instability and slow drift of the XFEL beam) which in combination with the KB focusing optic translates in fluctuation of the focal spot size and location. For the silica gel sample, a range of variation of the speckle size was measured to be [159,200] μm and [101,142] μm in the horizontal and vertical directions, respectively. Those for the gold nanoparticles were [66,107] μm for the horizontal direction and [66,95] μm for vertical direction (Supplementary Fig. S2).

Figure 2(c) shows the distribution of the speckle size *S* in the unit of pixel size by assuming an ellipsoidal speckle shape as:

2 $$S=\frac{\sqrt{(\pi /4)\times h\times v}}{({\rm{pixel}}\,{\rm{size}})}$$

where *h* and *v* are the horizontal and vertical FWHM speckle sizes, respectively. The histograms of *S* for both samples are presented in Fig. 2(c). The difference in overall speckle size between the two samples can be explained by the fact that both samples were measured at two different times, which could result in differences in the X-ray optical path to the sample.

**Figure 3:**
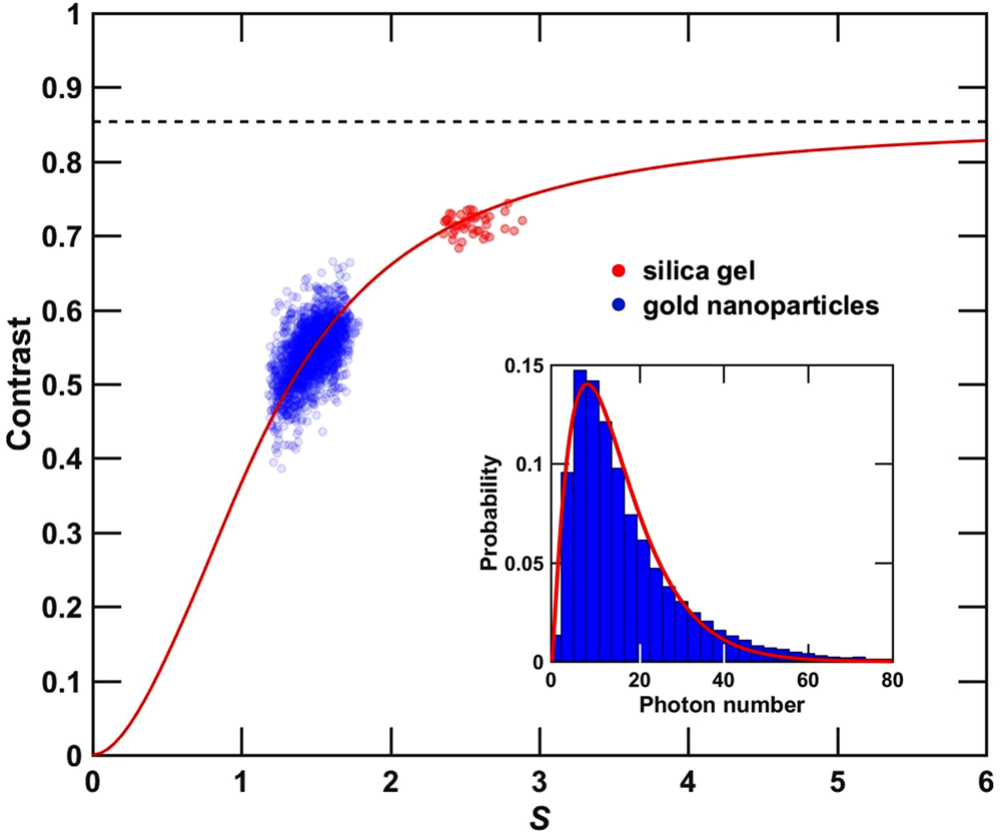
Contrast determination when considering experimental configuration. (Inset) Example of a single shot intensity histogram from the gold nanoparticle samples for pixels contained in the ROI and its fit using Eq. (1) as indicated by the solid line. (Main Figure) Measured contrast *β*_measure_ from many single-shot speckle patterns as a function of *S*, as defined in Eq. (2), are displayed for both the silica gel (red) and the gold nanoparticle (blue) samples. The red line shows the fit with Eq. (3). The dashed line indicates the value of the asymptotic behavior of Eq. (3) a guide to the eye and indicates *β*_intrinsic_ = 0.85 ± 0.06.

The inset of Fig. 3 shows the intensity histogram within the ROI of a single-shot scattering image from the gold nanoparticle sample. The solid line indicates the results of the fit using Eq. (1) yielding *M* = 3.43 ± 0.59 (i.e. *β*_measure_ = 0.55 ± 0.05). Figure 3 displays *β*_measure_ as a function of *S*, as obtained from each single shot speckle pattern for both samples. The data presented in Fig. 3 consist of 43 shots for the silica gel and 1572 for the gold nanoparticles. To factor in the experimental conditions on the measured contrast *β*_measure_, the contribution of the detector spatial averaging related to the ratio between the finite speckle size and the detector pixel size is considered. The contrast correction to *β*_measure_ is provided by^31,32^:

3 $${\beta }_{{\rm{m}}{\rm{e}}{\rm{a}}{\rm{s}}{\rm{u}}{\rm{r}}{\rm{e}}}(w)={\beta }_{{\rm{i}}{\rm{n}}{\rm{t}}{\rm{r}}{\rm{i}}{\rm{n}}{\rm{s}}{\rm{i}}{\rm{c}}}\cdot {[(\frac{2}{{w}^{2}}){\int }_{0}^{w}d\xi (w-\xi )\frac{{{\rm{s}}{\rm{i}}{\rm{n}}}^{2}(\xi /2)}{{(\xi /2)}^{2}}]}^{2}$$

with $w=2\pi /S$ where $S$ is the speckle size in units of pixel size as defined in Eq. (2) and ${\beta }_{{\rm{intrinsic}}}$ is the intrinsic speckle contrast free from any spatial averaging. The solid line in Fig. 3 is a non-linear least squares fit of ${\beta }_{{\rm{measure}}}$ using Eq. (3) and yields ${\beta }_{{\rm{intrinsic}}}$ = 0.85 ± 0.06 (as indicated by the dashed line).

In the present case, the experimental conditions to observe the full transverse coherence were not optimized. However, we were able to extract the intrinsic contrast for the PAL-XFEL beam by considering the spatial averaging of the speckle patterns and introducing the correction for the finite pixel size of the detector for that sample-detector distance.

Finally, we discuss how the longitudinal coherence and pulse duration are obtained from the analysis of the intensity distribution of subsequent X-ray pulses. Due to electron beam shot noise, which is inherent to the SASE process, a single XFEL pulse carries multiple temporal modes; the width of these represents the effective coherence time of the radiation. This spectral property of the light translates into fluctuations in the output beam intensity, which follows the Gamma density distribution:

4 $$p(I)=\frac{{M}^{M}}{{\rm{\Gamma }}(M)}{(\frac{I}{\langle I\rangle })}^{M-1}\frac{1}{\langle I\rangle }\exp (-M\frac{I}{\langle I\rangle })$$

**Figure 4:**
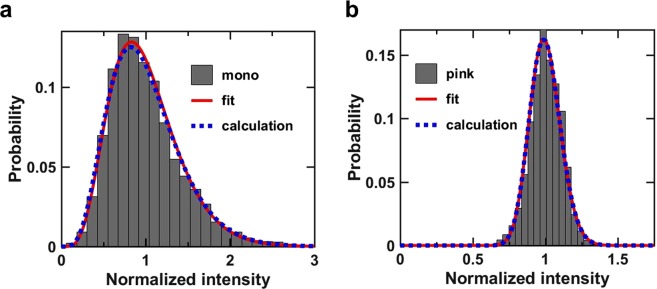
Intensity histograms for monochromatic and pink beam. (**a**) Histogram of the normalized shot-to-shot intensities for the monochromatic beam, its fit with Eq. (4) as indicated by the solid line (in red), and the calculation from the normalized variance by the dotted line (in blue). (**b**) Same histogram, the fit, and the calculation for the pink beam. The numbers of longitudinal modes obtained from the fits are 6.0 ± 0.4 and 90.0 ± 7.2 for the monochromatic and pink beam, respectively. Those calculated are 5.6 and 89.5 for the monochromatic and pink beam, respectively.

Note that the intensity distribution in a single-shot coherent X-ray scattering pattern follows the negative binomial distribution but the total integrated intensity from each single-shot satisfies the Gamma density distribution function for the linear FEL regime^33^. Analogous to the speckle transverse mode analysis, we extract the number of temporal modes by analyzing the distributions of intensity fluctuations^25^. In our experiment, we measured series of X-ray pulse intensities with and without a Si (111) Double Crystal Monochromator (DCM) which allowed comparing the statistical behaviors between the monochromatic and the pink (i.e. unfiltered) beam as displayed in Fig. 4(a,b) respectively. The fits of the distributions with Eq. (4) yield a mean number of temporal modes of <*M*_*t*_> = 6.0 ± 0.4 and 90.0 ± 7.2 for the monochromatic and the pink beam, respectively. In fact, the XFEL pulses measured in this study was in the saturation regime. However, Eq. (4) is valid since <*M*_*t*_> is much larger than 1, when the Gamma distribution of shot to shot pulse intensities approaches to a Gaussian distribution with a relative rms fluctuation given by $1/\sqrt{{M}_{t}}$. The calculated *M*_*t*_ from the normalized variance ${\rm{\sigma }}$ with the relation *M*_*t*_ = 1/$\,{\rm{\sigma }}\,$^2^ is also shown in the dotted lines in Fig. 4 (a,b) for comparison. They are 5.6 and 89.5 for the monochromatic and pink, respectively, and are well agreed with the fits.

Using the mean spectral bandwidth for the DCM monochromatic beam as Δ*E/E* = 1.36 × 10^−4^ and for the pink beam as 0.2%, we can estimate the coherence time τ_c_ using the relation below^22^:

5 $${\tau }_{c}=\frac{\lambda }{c}\frac{0.66}{({\rm{\Delta }}E/E)}$$

This yields τ_c_ = 2.06 fs and 0.14 fs for the monochromatic and pink beam, respectively. The pulse duration <T_p_> is estimated by <T_p_> = *M*_*t*_τ_c_. Our result thus implies that the average pulse duration at 9.73 keV at the PAL-XFEL is <T_p_> = 12.6 ± 1.0 fs. Note that this value can be underestimated since Δ*E/E* = 0.2% for the pink beam includes a broadening due to the energy chirps in the electron beam.

## Conclusions

We present the first characterization of the coherence properties in the hard X-ray regime of the PAL-XFEL. Our results indicate strong variations in the focused beam size on a pulse-to-pulse basis. After correcting for the effects of detector spatial averaging on the speckle contrast, we obtained an intrinsic speckle contrast *β*_intrinsic_ = 0.85 ± 0.06 at 9.73 keV. This confirms the nearly full transverse coherence of the PAL-XFEL beam. By analyzing the intensity distribution of subsequent X-ray pulses with and without a monochromator, we characterized the longitudinal coherence and provide a measurement of the pulse duration of the PAL-XFEL. We note that variations of the electron beam parameters (i.e. electron energy, bunch compression, undulator length, SASE vs. Seeding, etc.) can influence these coherence and pulse duration measurements. It would, therefore, be of great interest to extend both measurements as a function of the systematic variations of the XFEL operational parameters.

## Methods

### Sample preparation

Two samples were used: (i) a colloidal silica gel (S-chemtech) filled inside a quartz glass capillary consisting of colloidal silica spheres with a mean diameter of 50.4 ± 10.6 nm, dispersed in deionized water at a volume fraction of 3.5 vol%, (ii) 101 ± 11.8 nm gold nanoparticles (nano Composix) prepared in water at 1 vol% volume fraction and subsequently dried on a 12.5 μm thick Kapton film.

### Experimental configuration

The experiment was conducted at the NCI (Nano Crystallography and Imaging) end-station of PAL-XFEL^29,30^. The electron beam energy and bunch charge were 8.558 GeV and 140 pC, respectively. X-ray pulses with a mean photon energy of 9.73 keV were delivered from 20 undulators (each consisting of 5 m long undulator segments) to the experimental hutch at a repetition rate of 10 Hz. X-rays were focused at the sample position using KB mirrors.

The sample was translated after each X-ray pulse to avoid sample damage. The scattering signals from the sample were collected with a MPCCD 1 M detector which active sensor area consists of 1024 × 512 pixels with a pixel size of 50 μm × 50 μm^34^. The detector was placed 3.8 m downstream the sample; such that the speckle patterns can be resolved by the detector pixel elements.

## Supplementary information

Supplementary Information

## Data Availability

The data reported in this paper are available upon request. All code is also available upon request.
