# Supplementary material for: Coherence and pulse duration characterization of the PAL-XFEL in the hard X-ray regime
Source: Sci Rep. 2019 Mar 1;9:3300. doi: 10.1038/s41598-019-39765-3 (PMC6397240; doi:10.1038/s41598-019-39765-3)
Supplement: Supplementary file 1 — Supplementary Information [file 41598_2019_39765_MOESM1_ESM.docx]

Supplementary Information: Coherence and pulse duration characterization of the PAL-XFEL in the hard X-ray regime

Kyuseok Yun^1^, Sungwon Kim^1^, Dongjin Kim^1^, Myungwoo Chung^1^, Wonhyuk Jo^2^, Hyerim Hwang^2^, Daewoong Nam^3^, Sangsoo Kim^3^, Jangwoo Kim^3^, Sang-Youn Park^3^, Kyung Sook Kim^3^, Changyong Song^4^, Sooheyong Lee^2^, Hyunjung Kim^1^

^1^Department of Physics, Sogang University, Seoul 04107, Korea

^2^Korea Research Institute of Standards and Science, Daejeon 34113, Korea

^3^Pohang Accelerator Laboratory, Pohang 37673, Korea.

^4^Department of Physics, Pohang University of Science and Technology, Pohang 790-784, Korea

**Figure S1. Size determination of the silica gels and gold nanoparticles.**

Average radial intensities I(Q) of the Small Angle X-ray Scattering signal as a function of Q (symbol) are shown for (a) silica gel and (b) gold nanoparticles. This was performed by first averaging all single-shot speckle patterns and then done an azimuthal average. The fit is carried out on the high Q-range of the measured data, where there is no contribution from the static structure of the sample if present. It uses the convolution of a sphere form factor and a size distribution function. By assuming that the colloidal size polydispersity distribution is a Gaussian distribution, I(Q) is described by:

$I\left( Q \right)=I_{0}\int_{0}^{\infty} \frac{1}{\sigma\sqrt{2\pi}}\exp\left[ -\frac{\left( R-\bar{R} \right)^{2}}{2\sigma^{2}} \right]{V(R)}^{2}\left[ \frac{3J_{1}(QR)}{QR} \right]^{2}dR$,

where $J_{1}$ is the Bessel function of the first kind, $\bar{R}$ the averaged radius, and $\sigma$ the standard deviation of the size distribution. The solid line displays the result of the fit and indicates that the diameter of silica spheres in the gel is 50.4±10.6 nm and 101±11.8 nm for the gold nanoparticles.

**Figure S2. Histograms of the distribution of speckle size.**

(a) Histogram of the distribution of *h*, the speckle size in the horizontal direction. The horizontal size *h* spreads from 159 to 200μm for the silica gel sample and from 66 to 107μm for the gold nanoparticles. (b) Histogram of the distribution *v*, the speckle size in the vertical direction. The vertical size *v* varies from 101 to 142μm for the silica gel sample and spreads from 66 to 95 μm for the gold nanoparticles. Both graphs are plotted on the same scale for comparison.
